# Toward accurate vaginal microbiome profiling: protocol, bioinformatics, and core microbiota characterisation

**DOI:** 10.1007/s10815-025-03509-2

**Published:** 2025-05-29

**Authors:** Isabella M. Davidson, Elham Nikbakht, Larisa M. Haupt, Paul J. Dunn

**Affiliations:** 1 Faculty of Health Sciences & Medicine, Bond University, 14 University Drive, Robina QLD 4226, Gold Coast, Australia; 2 Stem Cell and Neurogenesis Group, Genomics Research Centre, Centre for Genomics and Personalised Health, School of Biomedical Sciences, Queensland University of Technology (QUT), 60 Musk Ave., Kelvin Grove, QLD, Brisbane, 4059 Australia; 3 ARC Training Centre for Cell and Tissue Engineering Technologies, Queensland University of Technology (QUT), Brisbane, Australia; 4 Max Planck Queensland Centre for the Materials Sciences of Extracellular Matrices, Queensland University of Technology (QUT), Brisbane, Australia

**Keywords:** Community state types (CSTs), Nanopore sequencing, Vaginal microbiome, *Lactobacillus*, Bioinformatics, 16S rRNA sequencing

## Abstract

**Purpose:**

Rising demand for assisted reproductive technologies (ART) with limited improvements in success rates has driven interest in the impact of the vaginal microbiome on fertility outcomes. In order to fully examine the relationship between the vaginal microbiome and fertility outcomes, methodologies and technological developments must be standardised and benchmarked to provide the most accurate assessment of microbial population representation.

**Methods:**

This study sought to investigate the utility of 16S sequencing and bioinformatic approaches using nanopore sequencing to characterize core vaginal microbiota in a healthy Australian cohort of reproductive-age women.

**Results:**

Optimisation and comparison of different PCR strategies for whole 16S amplification was undertaken, along with the generation of bioinformatic analysis strategies. Initial qPCR identified the 27F-YM (MIX) primer as the most sensitive for *C. trachomatis*. However, nanopore sequencing revealed no detectable *C. trachomatis* across all six samples. Among the bioinformatic tools, Porechop with NanoCLUST most accurately identified microbial presence. Community state type (CST) I—characterised by *Lactobacillus crispatus* dominance—was identified as the most common CST (66%), aligning with patterns of a healthy vaginal microbiome.

**Conclusion:**

The findings highlight a *Lactobacillus*-rich microbiome as the most common among healthy females; however, further refinement—potentially through a metagenomics approach—is recommended to address 16S rRNA primer limitations to enable improved accuracy of microbial detection for the vaginal microbiome.

**Graphical Abstract:**

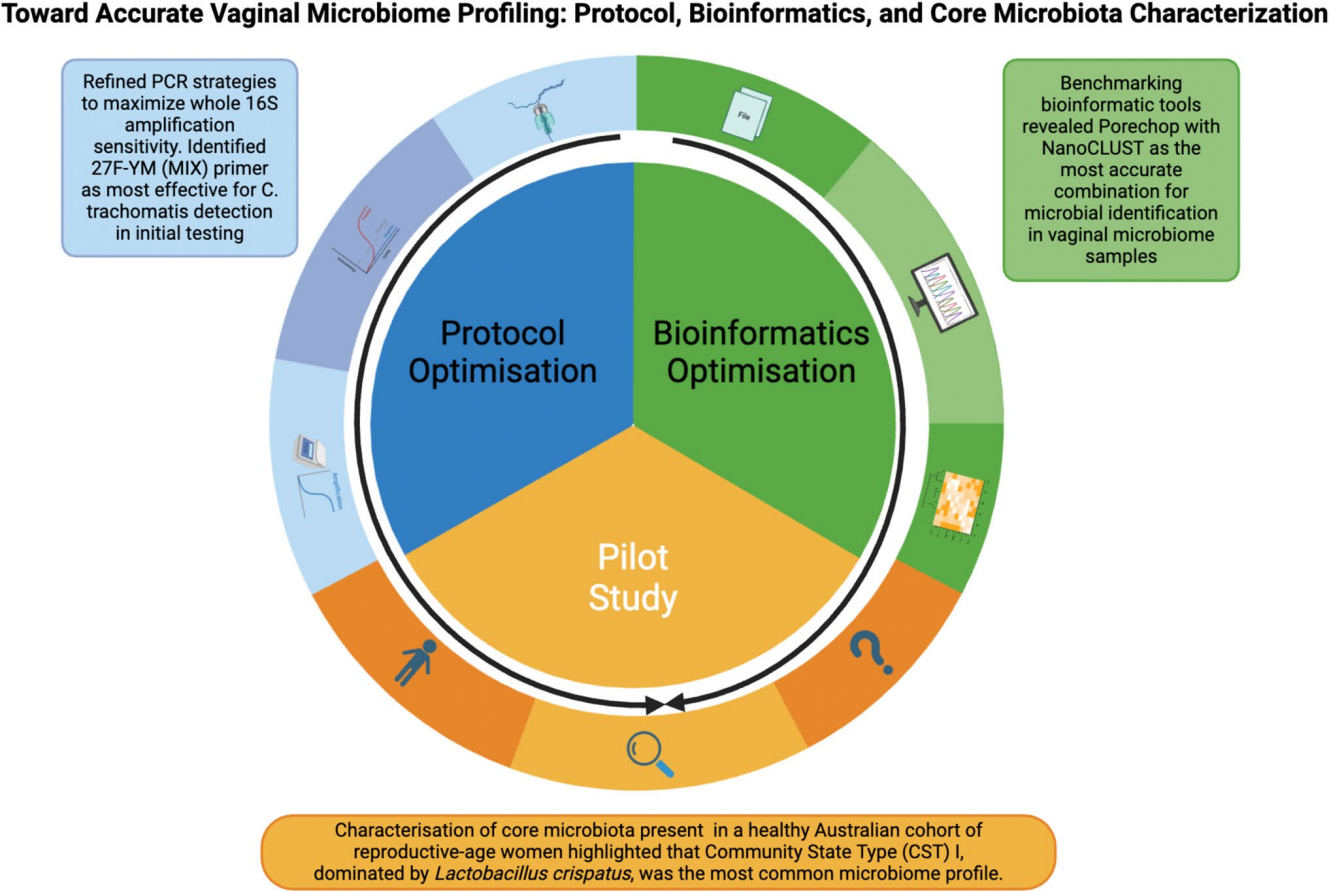

**Supplementary Information:**

The online version contains supplementary material available at 10.1007/s10815-025-03509-2.

## Introduction

The use of fertility treatment technologies such as in vitro fertilisation (IVF) and intracytoplasmic sperm injection (ICSI) has steadily climbed over the last 10 years, leading to increased interest in understanding the cause of infertility [1]. While factors such as weight, hormone levels, and even diet have been shown to influence female fertility, a plethora of other factors have also been postulated to contribute to the relatively low success rates surrounding fertility treatments [2, 3]. One such emerging area of interest is the impact of microbes that inhabit the female reproductive tract—in particular the vagina—with their influence on fertility yet to be thoroughly investigated.

The vaginal microbiome is a dynamic environment typically dominated by a homogenous variety of *Lactobacillus* species—such as *Lactobacillus crispatus*, *Lactobacillus iners*, *Lactobacillus gasseri*, and/or *Lactobacillus jensenii*—which promote homeostasis in the vagina whilst out-competing pathogenic microbes [4–6]. The emergence of the classification system community state types (CSTs) has enabled the importance of *Lactobacillus spp.* within the vaginal microbiota to be explored in relation to reproductive outcomes. Microbiomes are characterised as one of five CSTs (I, II, III, IV, and V) based on the dominant microbe or lack of *Lactobacillus spp*. dominant microbe (Table 1) [7].

**Table 1:** Dominant microbes present for each community state type with associated pregnancy favourability and overall diversity [7]

Community state type (CST)	Dominant microbe	Favourability for healthy pregnancy environment	Diversity
I	*Lactobacillus crispatus*	Extremely favourable	Low
II	*Lactobacillus gasseri*	Favourable	Low
III	*Lactobacillus iners*	Demonstrates conflicting favourability	Low
IV	No singular dominant species. Majority facultative and anaerobic bacteria	Associated with poorer reproductive outcomes	High
V	*Lactobacillus jensenii*	Favourable	Low

*Lactobacillus spp.* organisms have been shown to be favourable for a healthy pregnancy environment, where a *Lactobacillus*-dominated environment produces more successful results following in vitro fertilisation embryo-transfer (IVF-ET) [8, 9]. Conversely, vaginal microbiomes deemed to be dominated by bacterial vaginosis-associated microbes, such as *Gardnerella vaginalis* and *Atopobium vaginae*, have been correlated with negative reproductive outcomes [10]. Additionally, a correlation between abnormal vaginal microbiota and reduced clinical pregnancy rates has been identified in women undergoing IVF [11, 12]. As such, a vaginal microbiome consisting mainly of *Lactobacillus* has an increased likelihood of ART success.

Despite advancements in understanding the vaginal microbiome and fertility outcomes, substantial technical challenges in accurately assessing the vaginal microbiome remain. Methods to evaluate the vaginal microbiome have shifted from traditional culture-based approaches to molecular-based techniques such as PCR and 16S rRNA sequencing following the advancements of next-generation sequencing (NGS) technologies [13, 14]. NGS approaches such as nanopore sequencing allow for high-throughput information, sample pooling, and remarkable depth for species-level identification. However, primers commonly utilised for whole 16S rRNA sequencing exhibit significant difficulties in the assessment of accurate microbial population representation through underestimation or failure to recognise *C*. *trachomatis* and overestimation of *L*. *iners* [15, 16]. The inability to detect *C*. *trachomatis,* associated with increased risk of implantation failure and pregnancy loss in women undergoing fertility treatment, is detrimental to studies investigating the vaginal microbiome and its influence on ART outcomes [9, 14]. The presence of *L*. *iners* has also been shown to influence the outcome of fertility treatments, with its increased abundance associated with increased success rates for embryo implantation [16].

Current post-sequencing challenges of the rapidly developing nanopore platform include the lack of standardised, nanopore-compatible bioinformatic tools and pipelines [17]. With multiple requirements for further improvement, benchmarking, and design of standardised protocols and pipelines for microbial studies, studies comparing existing pipelines and tools available for custom pipelines and workflows remain limited [17–19]. This is in part driven by the increased error rate associated with long-read technologies, which has to date overshadowed the substantial possibilities of long-read sequencing for exploring complex bacterial communities via a real-time, high-throughput, and portable method [18].

The clinical impact of these methodological issues includes inaccurate diagnosis and treatment due to clinical misinterpretations, potentially causing harm to the patient or rendering treatments for an imbalance of microbes (dysbiosis) ineffective [12, 14]. Therefore, it is critical to design and utilise methodologies that define and represent microbiota accurately for subsequent analyses and interpretation. It is also essential to establish an accurate baseline of a healthy vaginal microbiome across populations before assessing the influence of the vaginal microbiome on fertility outcomes. This study aimed to optimise and refine a 16S rRNA sequencing approach with corresponding bioinformatic methodologies to enable accurate representation and analysis of microbial populations using Nanopore sequencing technology.

## Materials and methods

**Fig. 1:**
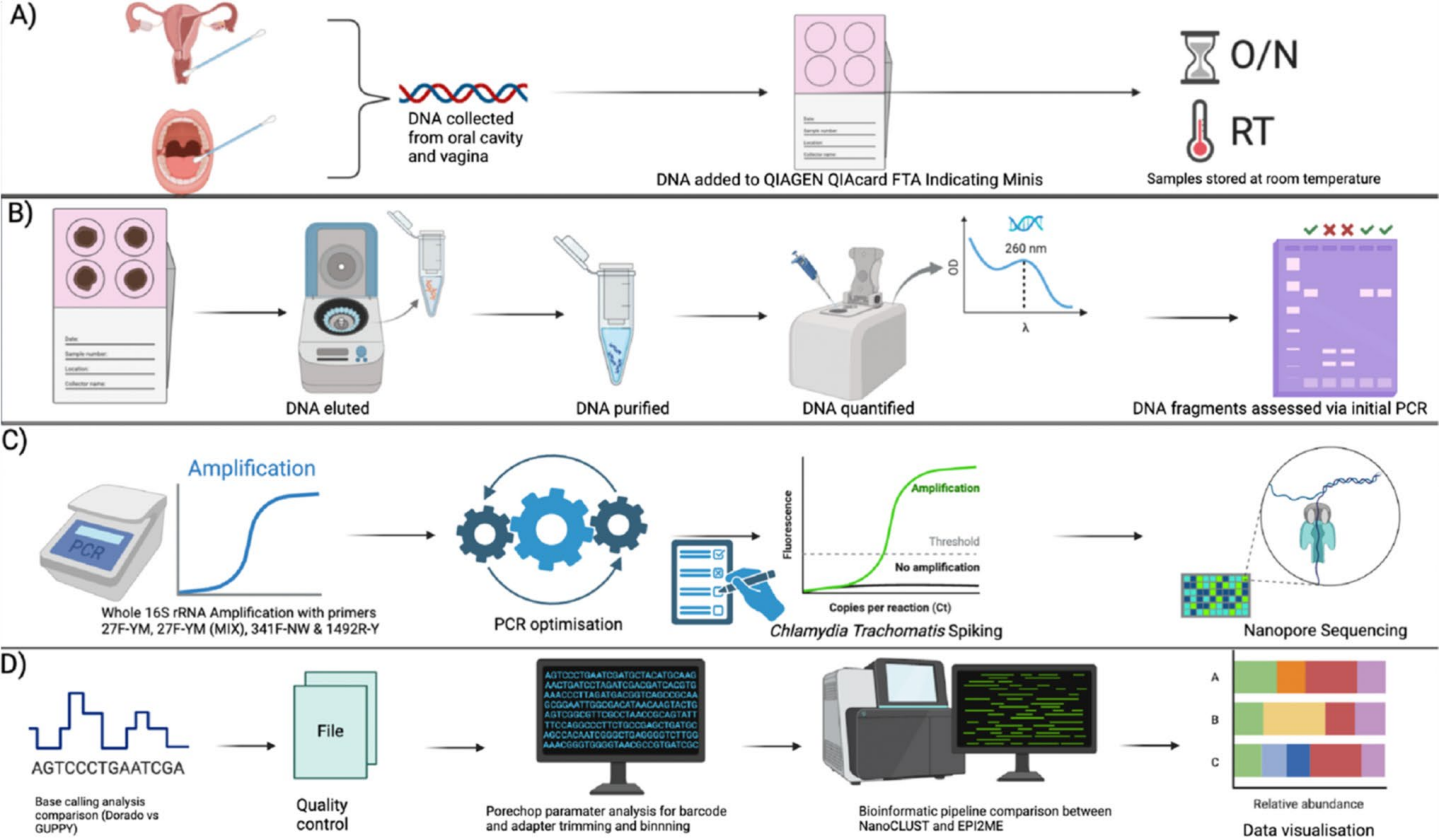
Representation of the full study design undertaken where **A** illustrates sample collection, addition of collected DNA onto QIAcards and DNA Storage. **B** DNA elution, purification and quantification as well as initial assessment of DNA fragments. **C** Whole 16S rRNA amplification of DNA utilising all primers, PCR optimisation, *C. trachomatis* spiking, and nanopore sequencing. **D** Base calling comparison, quality control assessment, Porechop adapter and barcode trimming and binning comparisons, NanoCLUST vs EPI2ME bioinformatic pipeline analysis, and data visualisation

The research received ethics approval from the Bond University Ethics Committee (PD00072). All participants signed a written consent form provided before sample collection. A figure of the full study design is presented in Fig. 1.

### Patient cohort, sample collection and storage

#### Sample collection and storage of oral microbiome samples

Three oral microbiome samples were collected via QIAGEN foam swabs and added onto QIAGEN QIACard FTA Indicating minis within the circle provided. Each participant provided a ‘fasting’ and ‘fed’ sample, resulting in a total of six samples. To ensure consistency, participants refrained from eating or drinking 30 min prior to collecting the ‘fasting’ sample. Following the first swab, participants consumed a Yakult probiotic drink, and after a five-minute wait, a second ‘fed’ sample was collected. In the absence of vaginal samples, oral swabs were initially used to assess the efficacy of laboratory methodologies in detecting microbial communities from samples expected to have an increased variety and number of microbes. The Yakult probiotic drink was selected to introduce more *Lactobacillus* spp., with the aim to more closely ‘mimic’ the vaginal microbiome [5, 6].

#### Participant selection and sample collection and storage of vaginal microbiome samples

Participants (*n* = 12) were pre-menopausal females aged between 18 and 55 with no currently known sexually transmitted infections (STIs), no current directed antibiotic treatment, and not currently pregnant. Once participants were selected based on selection criteria, no additional personal, survey, or medical details were collected. Detailed instructions were provided and each participant self-collected swab samples using a QIAGEN sterile foam swab. Briefly, participants were instructed to insert the swab ~ 5 cm (2 inches) into the vaginal opening, where it would be rotated against the wall of the vagina for approximately 15 s. Once completed, participants were instructed to remove the swab and press onto the FTA QIAcard Indicating Mini for sample collection. The cards were collected and stored at room temperature prior to DNA elution.

#### Bioinformatic comparison sample selection

Alongside oral microbiome samples, short- and long-read vaginal microbiome 16S sequencing data were obtained from the European Nucleotide Archive with the accession number PRJEB53337.

### Wet laboratory optimisation of 16s rRNA sequencing

#### DNA extraction

DNA was eluted from QIACards following the QIACard Elute Buffer Handbook—for elution of nucleic acids from QIACard FTA elute formats, with a protocol of elution of nucleic acids derived from biological fluids from QIACard FTA elute formats [20]. Briefly, seven punches of the FTA card were added to a microcentrifuge tube containing 400 µl elute buffer, repeated several times to wash samples before the addition of 10 µl proteinase K and elute buffer solutions, followed by incubation at 60 °C for 25 min and the sample mixed at 1000 rpm on a vortex for proteinase K digestion. Proteinase K was inactivated by raising the temperature to 95 °C for 5 min at 1000 rpm in a heat block with vortex, and punches were removed from the now-eluted DNA. DNA was then quantified and a separate working stock of eluted DNA at 20 ng/µl was prepared for downstream processing of all samples. Following extraction, DNA was stored at − 20 °C, and working stock solutions were stored at 4 °C.

#### PCR conditions and optimisation

Tailed primers containing the required sequence for Oxford Nanopore Technologies (ONT) amplicon outputs—27 F-YM, 27 F-Bor, 27 F-Bif, 27 F-Chl, 341 F-NW, and 1492R-Y (Table 2)—were diluted from a 100 µM stock to a 5 µM working solution using the Integrated DNA Technologies specification sheet and nuclease-free water. A 27 F-YM (MIX) was created by combining primers 27 F-YM, 27 F-YM_Bif, 27 F-YM_Bor, and 27 F-YM_Chl at a 4:1:1:1 ratio, as detailed in Wee et al. [21].

**Table 2:** Nucleotide sequences for each tailed ONT primer used throughout PCR (27 F-YM, 341 F-NW, 27 F-YM_Bor, 27 F-YM_Bif, 27 F-YM-Chl, 27 F-YM (MIX), and 1492R-Y (tailed sequence underlined) and ONT required tailed forward and reverse sequences

Primer	Sequence
27 F-YM	5′–TTTCTGTTGGTGCTGATATTGCAGAGTTTGATYMTGGCTCAG–3′
341 F-NW	5′–TTTCTGTTGGTGCTGATATTGCCCTACGGGNGGCWGCAG–3′
27 F-YM_Bor	5′–TTTCTGTTGGTGCTGATATTGCAGAGTTTGATCCTGGCTTAG–3′
27 F-YM_Bif	5′–TTTCTGTTGGTGCTGATATTGCAGGGTTCGATTCTGGCTCAG–3′
27 F-YM_Chl	5′–*T*TTCTGTTGGTGCTGATATTGCAGAATTTGATCTTGGTTCAG–3′
27 F-YM (MIX)	4:1:1:1 of 27 F-YM (4), 27 F-YM_Chl (1), 27 F-YM_Bif (1), and 27 F-YM_Bor (1)
1492R-Y	5′–ACTTGCCTGTCGCTCTATCTTCGGTTACCTTGTTAYGACTT–3′

The first round of amplification was performed using all primers and a total reaction volume of 25 µl, comprised of 12.5 µl 2X AllTaqMM, 1 µl each primer, and 8.5 µl dH_2_0 alongside 2 µl of DNA template per reaction. PCR amplification occurred with an initial denaturation at 94 °C for 4 min, followed by denaturation at 94 °C for 1 min, annealing at 48 °C for 30 s, elongation at 72 °C for 2 min, and final extension at 72 °C for 1 min with 25 total cycles, and finally a 4 °C hold.

#### PCR optimisation

PCR conditions were modified for primer 341 F-NW. The altered PCR conditions for 341 F-NW primer were: initial denaturation at 95 °C for 2 min, denaturation at 95 °C for 30 s, annealing at 53 °C for 40 s, elongation at 72 °C for 1 min, final extension at 72 °C for 10 min, with a total of 25 cycles and a 4 °C hold.

#### Chlamydia trachomatis qPCR

*Chlamydia trachomatis* was reconstituted following the Vircell Amplirun® *Chlamydia trachomatis* DNA control preparation protocol to a concentration of 13,000 copies/µl stock DNA sample. *Chlamydia* stock was then serially diluted at a 1:100 ratio to produce concentrations of 1300 copies/µl, 130 copies/µl, 13 copies/µl, and 0.13 copies/µl. A Q-PCR reaction was established using SSO SuperMix (Evageen) and 5 µl of each concentration in triplicate for all primers. PCR conditions for primers 27 F-YM and 1492R-Y, and 27 F-YM_CHL and 1492R-Y, included initial denaturation at 94 °C for 4 min, denaturation at 94 °C for 1 min, annealing at 48 °C for 80 s, elongation at 72 °C for 2 min, and final extension at 72 °C for 2 min with 35 total cycles. For primer pair 341 F-NW and 1492R-Y, conditions were: initial denaturation at 95 °C for 2 min, denaturation at 95 °C for 30 s, annealing at 53 °C for 1 min, elongation at 72 °C for 1 min, and final extension at 72 °C for 10 min, also totalling 35 total cycles. Following initial Q-PCR, a second was performed with all primers, including primer pair 27 F-YM (MIX) and 1492R-Y, diluted to a final concentration of 500 nM. The conditions were: initial denaturation at 98 °C for 2 min, denaturation at 98 °C for 30 s, annealing at 57.5 °C for 80 s, elongation at 72 °C for 1 min, and final extension at 72 °C for 10 min, with 44 total cycles. Throughout Q-PCR, cycling temperatures, times, and total cycles were systematically varied to optimise the amplification of *Chlamydia* DNA.

#### Chlamydia trachomatis spiking

A PCR reaction was prepared for all primer sets under conditions from the optimised protocol, incorporating 1 µl of 130 copies/µl *C. trachomatis* to each sample, giving a total volume of 26 µl. An additional spike was performed using only the 27 F-YM (MIX) and 27 F-Chl primers without DNA samples, containing *C. trachomatis* DNA. *C. trachomatis* at concentrations of 130 and 650 copies/µl (1300 halved) in triplicate for both primers, and amplification was performed as per the 27 F protocol.

A subsequent PCR employed triplicates of 27 F-YM_CHL and 1492R-Y, and 27 F-YM (MIX) and 1492R-Y primers using the same master mix preparation and adding 1 µl of 130 or 650 copies/µl using the previously optimised PCR conditions for AllTaq MasterMix (QIAGEN). A final PCR reaction was prepared for primer pairs 27 F-YM (MIX) and 1492R-Y, 27 F-YM and 1492R-Y, and 341 F-NW and 1492R-Y, with the addition of 1 µl of 650 copies/µl *C*. *trachomatis* DNA to oral microbiome DNA, resulting in a total reaction volume of 26 µl. The final reaction was performed using an optimised protocol for the 341 F-NW and 1492R-Y primer pair using the 27 F amplification protocol. All PCR products were analysed using either a QIAxcel or gel electrophoresis to assess if amplicons were present. All PCR reactions included a negative control containing 23 µl master mix and 2 µl dH_2_0 to assess for DNA contamination.

#### PCR optimisation for C. trachomatis spiking

PCR conditions were initially optimised by increasing the number of cycles from 25 to 44, using a master mix prepared with 130 copies/µl to increase the likelihood of amplification. To minimise primer dimer formation, a final concentration of 0.25 µM for each primer was prepared and compared to a 5 µM primer concentration along with the addition of 130 copies/µl *C*. *trachomatis* DNA. PCR conditions were: initial denaturation at 95 °C for 3 min, denaturation at 95 °C for 15 s, annealing at 60 °C for 30 s, elongation at 68 °C for 90 s, final extension at 68 °C for 10 min, with 40 total cycles.

Subsequent PCR reactions utilised the 27 F-YM_CHL and 1492R-Y primer pair with final primer concentrations of 0.25 and 5 µM in combination with *C*. *trachomatis* DNA concentrations of 100, 500, and 1000 copies/µl added. The PCR conditions were: denaturation at 94 °C for 4 min, denaturation at 94 °C for 1 min, annealing at 48 °C for 30 s, elongation at 72 °C for 2 min, final extension at 72 °C for 1 min, with 40 total cycles. Identical master mix and DNA concentrations were applied to primer pairs 27 F-YM and 1492R-Y, 27 F-YM (MIX) and 1492R-Y, 27 F-YM_CHL and 1492R-Y, and 341 F-NW and 1492R-Y using PCR conditions of: initial denaturation 94 °C for 4 min, denaturation 94 °C for 1 min, annealing at 48 °C for 40 s, elongation at 72 °C for 1 min, final extension at 72 °C for 2 min with 39 total cycles. For the 341 F-NW and 1492R-Y primer pair, the following conditions were employed: initial denaturation at 95 °C for 2 min, denaturation at 95 °C for 30 s, annealing at 53 °C for 40 s, elongation at 72 °C for 1 min, final extension at 72 °C for 10 min.

Various primer concentrations (1, 2.5, 3, and 5 µM) were analysed, with each reaction containing 1 µl of primer and 1 µl of 130 copies/µl *C*. *trachomatis* DNA, utilising the most recent PCR conditions.

A final PCR reaction was prepared utilising an annealing temperature gradient for all primers (5 µM) with 1 µl 650 copies/µl DNA added per primer pair. The PCR conditions used were: initial denaturation at 94 °C for 4 min, denaturation 94 °C for 1 min, annealing gradient from 46 to 58 °C, elongation 72 °C for 2 min, final extension at 72 °C for 1 min, with 40 cycles. The annealing temperature gradient used was: well A = 58 °C, B = 57.2 °C, C = 55.7 °C, D = 53.4 °C, E = 50.6 °C, F = 48.3 °C, G = 46.8 °C, and H = 46 °C.

#### Library preparation

Whole 16S rRNA amplicon sequencing (regions V1–V9) was achieved through the use of 27 F-YM (MIX) and 1492R-Y, and 27 F-YM and 1492R-Y primer pairs, whereas sequencing of regions V3/V4–V9 was achieved via amplification using the 341 F-NW and 1492R-Y primer pair. Prior to PCR barcoding and ligation and following DNA quantification, 100 fmol solutions were created for each sample. ONT’s PCR barcoding ligation v14 protocol was followed for PCR barcoding and ligation with minor changes to the protocol [22]. The PCR barcoding protocol was adapted to the addition of 1 µl of the corresponding barcode, 15 µl of 100 fmol DNA, 7.5 µl 4X AllTaq Master Mix (QIAGEN), and 7.5 µl dH20, to a total reaction volume of 31 µl per sample. The PCR amplification protocol was also adapted to: initial denaturation at 93 °C for 3 min, denaturation at 93 °C for 30 s, annealing at 55 °C for 15 s, elongation at 68 °C for 1 min, final extension at 72 °C for 10 min, with 15 total cycles and a 4 °C hold. Samples utilising LongAmp Taq 2X Master Mix (New England Biolabs), where the PCR barcoding mix was comprised of 1 µl barcode, 12 µl LongAmp Taq 2X Master Mix, and 12 µl 100 fmol DNA, followed the protocol conditions of 95 °C for 2 min, 95 °C 15 s, 62 °C for 15 s, 65 °C for 1 min, and 65 °C for 10 min for 15 total cycles. Samples were ligated according to the manufacturer’s protocol, with 1 µg of pooled library prepared for end-prep. End-prep and adapter ligation clean-up was followed using the ligation sequencing kit (SQK-LSK114) manual with addition of a short fragment buffer. Following barcoding and ligation, DNA was sequenced on a Flongle flow cell r10 version (FLO-FLG114) with base calling off and a minimum read length set to 200 bp on an Oxford Nanopore Technologies MinION for 24 h or until the read plateaued.

### Bioinformatics comparison analysis

#### Pre-processing and quality control

Base calling was initially performed on oral microbiome samples directly on the MinION Mk1c sequencer using GUPPY software with the high-accuracy model (V6.5.7). Dorado, an open-source base caller for Oxford Nanopore sequencing reads, was used for simplex base calling and duplex base calling, utilising the model dna_r10.4.1_e8.2_260bps_hac@v4.0.0 (v0.8.2). After base calling was completed, BAM output files created from Dorado were converted from BAM to FASTQ files via the software samtools (v1.13) for downstream analysis. Prior to adapter trimming, each FASTQ file (generated data and Lüth et al. data) was subject to raw sequence data quality control using the analysis tools FastQC (v0.11.9) for singular files and MultiQC (v1.21) to summarise quality from the FastQC reports.

#### Adapter trimming and barcode demultiplexing

For each method of base calling, GUPPY or Dorado, two different parameters for adapter trimming and filtering were trialled in Porechop (v0.2.4) [23]. Specific and non-specific adapter and barcode sequences were compared via the specific method involving manually altering the Porechop adapters.py file to only allow for input adapter and barcodes sequenced. The non-specific method did not alter the file, leaving the provided sequences. All self-collected data was filtered into NanoCLUST (v1) with default parameters, with reads extracted from Lüth et al. unable to be processed by NanoCLUST using the default parameters. Therefore, NanoCLUST and Porechop parameters had to be configured to enable downstream analysis through less stringent criteria. Altered parameters included a barcode threshold of 70% (default 75%) and barcode diff of 2% (default 5%). Briefly, the barcode threshold is the match percentage of the barcode, and barcode diff is the percentage difference between two barcodes required to be binned, ultimately decreasing the stringency of barcode assignment and binning [23].

#### Read clustering and analysis

NanoCLUST and EPI2ME (v5.1.10) pipelines were compared for read clustering, consensus building, and microbial population output. Both pipelines were implemented using data that had undergone barcode demultiplexing and adapter trimming via Porechop. post-Porechop demultiplexing and trimming with a minimum 500 bp read length set for all samples.

Due to the stringent parameters of NanoCLUST, the polishing_reads parameter for raw long-read data extracted from Lüth et al. was lowered from 100 to 25. The adjustment was required for computational feasibility. Setting the polishing_reads parameter ultimately decreased the number of reads used in the polishing process and final assembly, which reduced computational load. A second run was performed with the same simplex base-called raw data but with the polishing reads parameter set to 25 for all samples to determine the impact of this change on taxonomic classification. While NanoCLUST has a built-in demultiplex feature, it was not utilised throughout this study. However, both the demultiplex via NanoCLUST and demultiplex via Porechop options were trialled using the simplex base calling of raw data to determine if any taxonomic classification differences arise from using NanoCLUST to trim, filter, and demultiplex or Porechop prior to the NanoCLUST pipeline.

In addition to NanoCLUST, Oxford Nanopore’s cloud-based analysis pipeline EPI2ME was also examined for all base calling data (simplex, duplex, and GUPPY) and raw nanopore reads obtained from Lüth et al. The platform includes a specific 16S workflow tailored to the error-prone long nanopore reads and the MinION sequencer [24]. Using demultiplexed reads, each sample was analysed through the 16S workflow using Kraken2 to classify, with a minimum read length of 500 bp and taxonomic rank species as specific parameters, with the default kept for the rest of the parameters.

Upon completion of analysis, EPI2ME provided a stacked bar chart of the top 9 most abundant taxids. Concurrently, the 20 most abundant taxids from each sample output from NanoCLUST were collated into one CSV. The file was arranged alphabetically, and column headers were changed numerically for each sample. Any duplicates were removed from the collated CSV file, and once complete, the file was ready for data visualisation.

#### Bioinformatic pipeline comparison criteria

Each bioinformatic tool and pipeline was evaluated based on their effectiveness in taxonomic identification and relative abundance demonstration. Initial comparison was performed using two different base calling software: GUPPY and Dorado. Within that comparison, Dorado duplex and simplex base calling methods were assessed via raw data quality and output in subsequent analyses. Following base caller comparison, Porechop adapter trimming was compared to determine whether inputting specific adapter and barcode sequences improved the accuracy of microbial detection and representation over default settings. Both comparisons utilised sequencing outputs from oral microbiome samples. FASTQ files obtained from Lüth et al. were processed through both specific and non-specific Porechop adapter parameters and compared to the paper’s findings to identify the most effective parameter for the tool. Additionally, the processed data was analysed using pipelines NanoCLUST and EPI2ME, with taxonomic output compared to the findings of Lüth et al. to determine which pipeline was the most accurate for taxonomic population representation.

#### Data visualisation

Data was visualised using RStudio (version 2023.09.1 + 494) in two ways: overall bacterial abundance via a heatmap and a stacked bar chart of the fraction of reads of the most abundant bacteria detected and classified. Use of the *pheatmap* library was employed for heatmap generation, and the grid library for primer presentation. In contrast, the libraries *readr*, *tidyr*, and *ggplot2* were implemented to create the required data frame and form a stacked bar chart.

## Results

### Primer sensitivity for *C. trachomatis* detection via qPCR amplification

Amplification of different 16S rRNA primer mixes was tested at various *C. trachomatis* concentrations to determine their effectiveness in detecting *C. trachomatis* in samples. The degenerate 27 F-YM (MIX) primer set showed the most consistent amplification of the *Chlamydia* standard, which amplified all concentrations, reaching RFU values of 900. The 27 F-YM and 27 F-YM_Chl primers showed partial amplification at 1300 and 130 copies/µl *Chlamydia* standard, whereas 0.13 and 13 copies/µl *Chlamydia* standard failed to amplify. The 341 F-NW primer failed to amplify the *Chlamydia* control at all concentrations.

### PCR product visualisation for all primers with Chlamydia trachomatis added

**Fig. 2:**
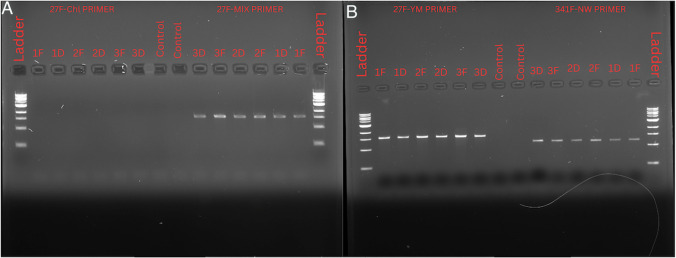
Gel electrophoresis results showing PCR amplification of oral DNA samples spiked with 130 copies/µl C. trachomatis using different primer sets. **A** Amplification using the 27 F-Chl primer (left) and 27 F-YM (MIX) primer (right). **B** Amplification using the 27 F-YM primer (left) and 341 F-NW primer (right). Each lane represents a DNA sample obtained under fasting (F) or post-Lactobacillus Drink (D). dH_2_0 Controls are also included for comparison. A 500 bp–10 kb DNA size ladder (New England Biolabs) is present on both sides of each gel for fragment size estimation

Amplification for each primer with 130 copies/µl *Chlamydia* standard to oral microbiome samples resulted in successful amplification of the 27 F-YM (MIX), 27 F-YM, and 341 F-NW primers (Fig. 2). In contrast, the 27 F-YM_Chl primers resulted in no DNA amplification.

### Sequencing outcomes

**Fig. 3:**
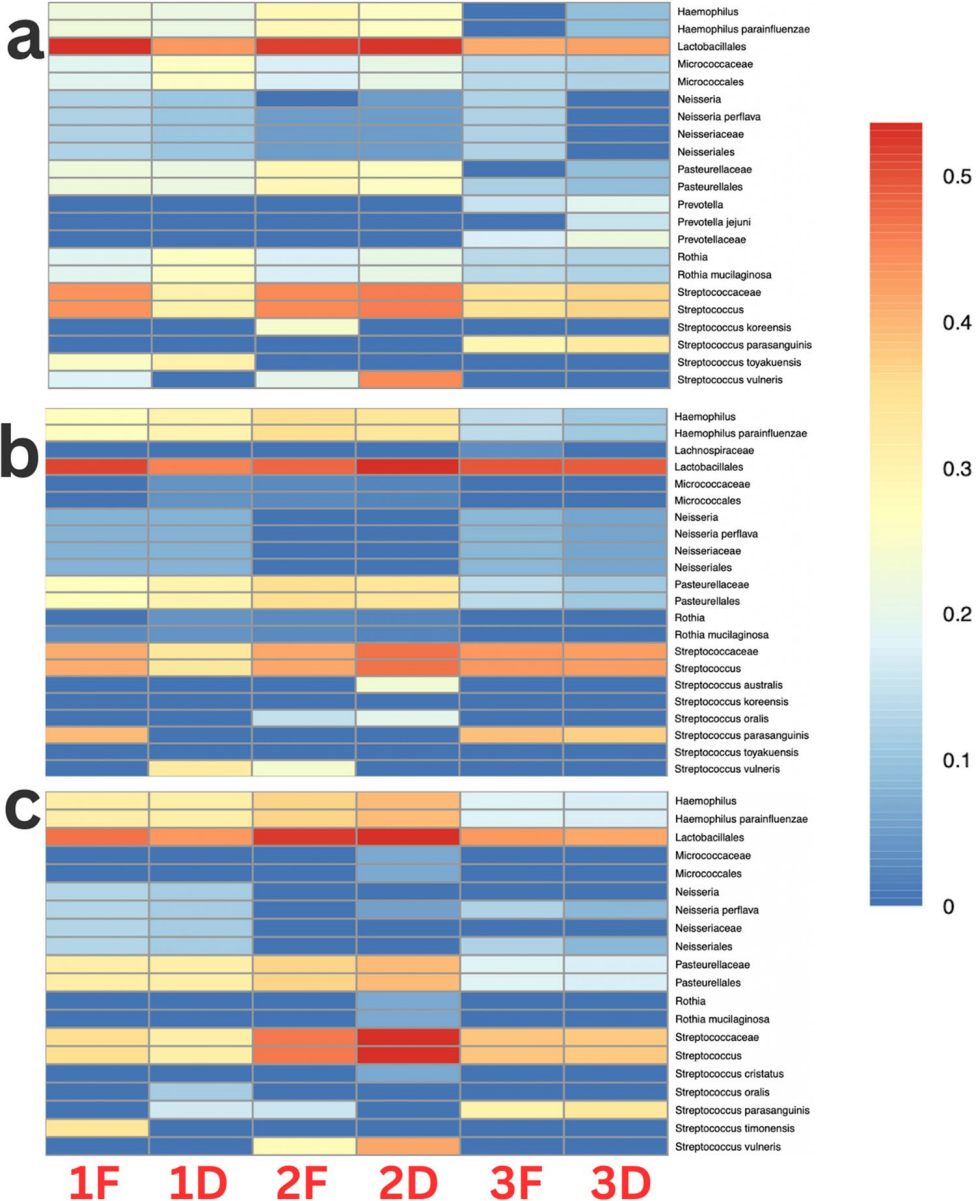
Heatmap representation of the most prevalent microbial species in oral microbiome DNA samples following nanopore sequencing with different primer sets. **a** 27 F-YM primer, **b** 341 F-NW primer, and **c** 27 F-YM (MIX) primer were used to amplify DNA from samples 1 F, 1D, 2 F, 2D, 3 F, and 3D, each spiked with 650 copies/µl *C. trachomatis*. The heatmap displays relative abundances, with colour intensity corresponding to the detected proportion of each microbial species (blue = low abundance; red = high abundance). Data was visualised using R Studio (rstudio-education.github.io)

All primer combinations failed to identify *Lactobacillus* at the species or genus level, even within oral samples with and without the addition of a *Lactobacillus*-containing drink (Yakult) (Fig. 3). All primer combinations classified to the order level *Lactobacillales* regardless of supplementation with a *Lactobacillus*-containing drink. Each primer demonstrated a slightly different relative abundance of *Lactobacillales* within the same samples, with only sample 2D demonstrating consistency across all three primers. Despite the addition of 650 copies/µl *Chlamydia* standard, all three primers failed to identify *C. trachomatis* within the samples tested.

### Bioinformatics comparison analysis

#### Relative evaluation of each base calling method, bacterial identification and abundance detection

The use of specific and non-specific Porechop adapter and barcode trimming and demultiplexing parameters revealed discrepancies in microbial detection, identification of bacterial species, and their relative abundance between the same bacteria within the dataset. Base calling software for non-specific and specific Porechop parameters showed a minimal difference, with slight variations in some singular bacterial abundance and/or identification across all three software options. Overall, only minor variations were observed between each base calling option, with different abundances identified in specific samples. However, results were generally consistent across all combinations of sample and software used (Supplementary Fig. 1S2).

#### Comparative analysis of bioinformatic pipelines NanoCLUST and EPI2ME using the Lüth et al. publicly available vaginal microbiome raw sequencing data

The bioinformatic pipelines NanoCLUST and EPI2ME were developed to handle nanopore sequencing data, to analyse microbial community data, and to assign taxonomies through read clustering and consensus building. Both pipelines produce output of taxonomic classification, the NanoCLUST data output providing a summary of all detected microbes and relative abundances exportable as a CSV file for downstream analysis. In contrast, the EPI2ME pipeline enables the user to analyse and visualise the data and displayed the top eight to nine most abundant microbes across levels of classification.

Data analyses revealed the utilisation of Porechop-specific adapter trimming and barcode binning parameters correctly matched the number of patients (*n* = 10), whilst the non-specific parameters for adapter trimming and barcode binning output resulted in two extra barcode bins representative of two additional ‘phantom patients’ (*n* = 12). The specific Porechop parameters coupled with NanoCLUST identified high-level concordance with the findings of Lüth et al. This is demonstrated with 8/10 samples showing similar species abundance when compared with 0/10 non-specific when coupled with NanoCLUST, 2/10 non-specific when Porechop analysis was paired with EPI2ME, and 5/10 when specific Porechop parameters were coupled with EPI2ME (Table 3) for analysis. Misclassification of different *Lactobacillus* spp. discordant to Lüth et al.’s findings were prevalent, primarily among microbes *L. gasseri* and *L. paragasseri*, and between *L. iners* and *L. crispatus*.

**Table 3:** Comparison of NanoCLUST and EPI2ME pipelines to Lüth et al. nearly full-length nanopore sequencing data for bacterial abundance with microbe names in order of highest relative abundance (top 3 most abundant)

Sample	(Lüth et al. 2022*)*	NanoCLUST		EPI2ME	
		Nonspecific	Specific	Nonspecific	Specific
1	*H. aegyptis*, *G. vaginalis*	*H. parainfluenzae*, *R. mucilaginosa*, *S. australis*	*H. Aegyptus, G. swidsiniskii*, *S. massiliensis*	Unknown, other, *L. gallinarum*	Unknown, *H. influenzae*, *H. aegyptius*
2	*L. crispatus and G. vaginalis*, *enterobacteriacaea*	*R. mucilaginosa*, *H. parainfluenzae*, *S. australis*	*L. crispatus*, *G. vaginalis*	Unknown, other, *G. vaginalis*	*G. vaginalis,* other, *L. crispatus*
3	*V. atypica*, *L. gasseri*, *Anaercoccus*	*S. cristatus*, *R. mucilaginosa*, *H. parainfluenzae*	*N. stercoris*, *L. gasseri*, *V. atypica*	Unknown, other, *H. aegyptius*	Unknown, other, *P. timonesis*
4	Preveotella, Clostriales, S. amnii	*H. parainfluenzae*, *R. mucilaginosa, S. australis*	*H. timonesis, M. lornae*, *S. sanguinegens*	Unknown, other, *L. iners*	Other*, P. timonensis*, *G. vaginalis*
5	*A.vaginae*, *L. crispatus*,* G. vaginalis*	S. *parasanguinis*, V. *dispar*, H. *parainfluenzae*	*L. iners, L. jensenii*, *U, parvum*	Unknown, other, *H. aegyptius*	Other, unknown, *G. vaginalis*
6	*L. crispatus*, *L. iners*, *enterobacteriacaea*	*S. parasanguinis*, *V. dispar*, *H. parainfluenzae*	*L. crispatus*, *L. iners*	Unknown, other, *G. vaginalis*	*L. crispatus, L. iners*, *L. gallinarum*
7	*L. gasseri, L. crispatus, Bifidobacterium*	*H. parainfluenzae*, *S. parasanguinis*, *S. vulneris*	*L. gasseri, B. breve*, *P. bivia*	Unknown, other, *G. vaginalis*	Other*, L. paragasseri, L. gallinarum*
8	*L. iners*, *L.crispatus,*	*S. parasanguinis*, *H. parainfluenzae*, *G. elegans*	*L. iners*	Unknown, other, P. *timonesis*	Unknown, *L. iners*
9	*G. vaginalis*, *S. anginosus*, *L. gasseri*	*S. vulneris*, *H. parainfluenzae*, *G. elegans*	*G. leopoldii*, *G. vaginalis*, *S. parasanguinis*	Unknown, other, *L. crispatus*	*G. vaginalis*, other, unknown
10	*L. gasseri*, *G. vaginalis*, *L. crispatus*	*H. parainfluenzae*, *S. vulneris*, *S. australis*	*L. gasseri*, *G. morbillorum*, *P. terephthalicicum*	Unknown, other, *L. crispatus*	Unknown, *L. paragasseri*, other
11		*S. parasanguinis*, *H. parainfluenzae*, *V. nakazawae*		Unknown, other, *H. influenzae*	
12		*S. parasanguinis*, *V. nakazawae*, *L. gingivalis*		Unknown, other, *L. paragasseri*	

#### Determination of core vaginal microbiota in a healthy population results

**Fig. 4:**
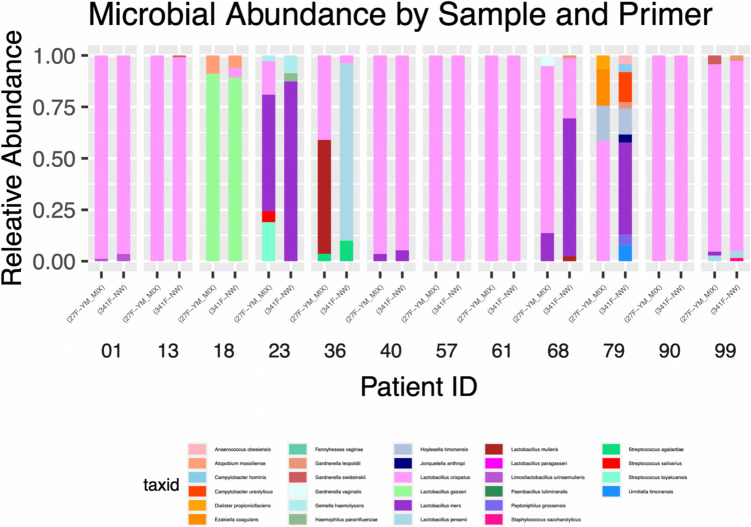
Stacked bar chart of bacterial relative abundance in vaginal microbiome samples using only two primer sets. The chart displays the relative abundance of bacterial taxa detected in vaginal (01, 13, 18, 23, 38, 40, 57, 61, 68, 79, 90, and 99) using 27 F-YM (MIX) and 341 F-NW primers. Each sample is grouped to facilitate direct comparison between primer sets. Colours represent different bacterial taxa, with proportions indicating their relative abundance in each sample. Data visualisation was performed using in R Studio (rstudio-education.github.io)

Sequencing analysis of collected vaginal microbiome samples identified concordant dominant microbes in 9/12 samples (Fig. 4). Samples 01, 13, 18, 40, 57, 61, 90, and 99 all showed extremely similar microbial abundance using both primers, with *L. crispatus* demonstrating dominance over samples 01, 13, 40, 57, 61, 90, and 99, with sample 18 demonstrating *L. gasseri* dominance. Sample 23 demonstrated the same dominant microbe (*L. iners*). However, the 27 F-YM_MIX primer demonstrated reduced relative abundance and identified microbes including *L. mulieris*, *L. crispatus,* and *L. gasseri* not identified using the 341 F-NW primer.

The remaining three samples (36, 68, and 79) were found to have differences in the abundance of *Lactobacillus spp*. Sample 68 showed similarities in abundance of the two major microbes identified (*L. crispatus* and *L. iners*), with their relative abundance shown to be inverse between both primers (27 F-YM_MIX primer displaying higher *L. crispatus* and 341 F-NW displaying higher *L. iners* abundance). Significant microbial discrepancies were also observed between samples 36 and 79, with different dominant microbes identified. Although a part of the same genus, analysis with primer 27 F-YM_MIX for sample 36 resulted in *L. mulieris* identified as the dominant microbe, whereas the 341 F-NW primer determined *L. jensenii* as the dominant microbe. Not only were the dominant microbes different across the two primers for the same sample, but the 27 F-YM_MIX primer displayed *L. crispatus* as the second most abundant microbe, whereas 341 F-NW demonstrated *L. jensenii* at over 80% of the microbial abundance, with *L. crispatus* comprising less than 10%. Sample 79 displayed *L. crispatus* dominance for the 27 F-YM_MIX primer, whilst 341 F-NW highlighted *L. iners* to be in large abundance. Whilst both primers detected a similar amount of *Hoylesella timonensis* in sample 79, the 27 F-YM_MIX primer illustrated the presence of *Diallister propionicifaciens* and *Ezakiella coagulans* at over 20%, while 341 F-NW showed *Campylobacter ureolyticus*, *Gardnerella leopoldii*, and *Anaerococcus obesiensis* in sample 79.

Further investigation of bacterial abundance within the samples was conducted to identify community state types, as well as to investigate whether discordant results influenced these classifications (Table 4). From the 9 concordant samples, CST I accounted for 7/9 samples, while the remaining samples were found to be comprised of CST II (sample 18) and CST III (sample 23). Discordant microbial dominance across both primers in samples 36, 68, and 79 also resulted in different CST classifications. These differences were due to variations in detection of *Lactobacillus* sub-species. Sample 36 demonstrated *L. jensenii* dominance (CST V) for the 27 F-YM_MIX primer, whilst *L. mulieris* was shown to be dominant using the 341 F-NW primer, with *L. crispatus* the next most dominant species. Interestingly, *L. mulieris* has not been attributed to a vaginal CST. Sample 79 demonstrated different dominant microbes, specifically *L. crispatus* for 27 F-YM_MIX (CST I), and *L. iners* for 341 F-NW (CST III). The discordance identified within sample 68 highlights two different CSTs with the 27 F-YM_MIX (CST I) and 341 F-NW (CST III).

**Table 4:**
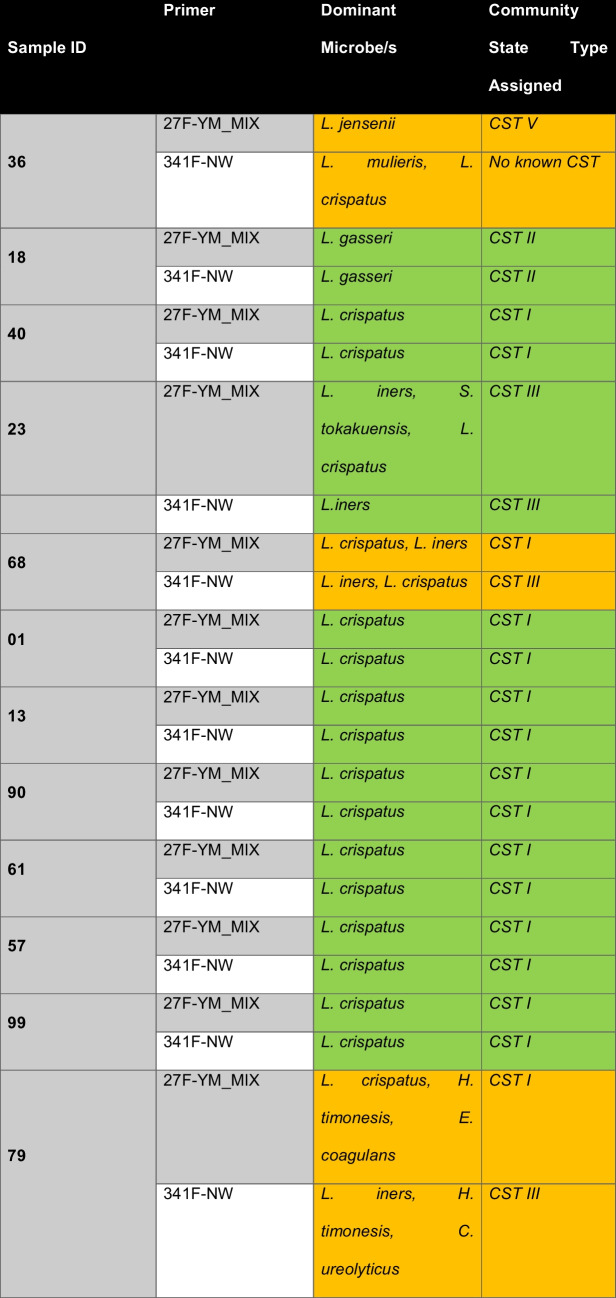
Most dominant microbes present in participant vaginal samples for primers 27 F-YM_MIX and 341 F-NW and the assigned community state type according to Moosa et al. with green indicating a match between primers and orange representing different community state type assignment for the same sample across different primers [7]

## Discussion

Methodological development and comparison of 16S rRNA sequencing techniques, such as primer design and bioinformatic considerations, highlight current limitations across microbial detection in control and sample (vaginal) microbiomes. In addition, the application of different bioinformatic tools influences microbial abundance representation. A key limitation in this study was the inaccuracy of microbial detection and representation due to nucleotide mismatches within whole 16S rRNA sequencing primers (27 F) [25]. While the 27 F-YM and 27 F-YM primer mix was suggested as a potential solution due to the addition of degenerate bases and species-specific sequences, challenges were still encountered with accuracy of microbial detection, in particular *C. trachomatis* [16, 25]. Although unable to sequence the entire 16S rRNA gene, the inclusion of a 16S V3 region primer was proposed to improve microbial detection accuracy, with improved identification of microbiomes such as *C. trachomatis* and *L. iners* [25].

Prior to completing nanopore NGS, template from *C. trachomatis* was added to oral microbiome samples to determine concentration sensitivity for amplification. Subsequently, these oral samples were sequenced to evaluate differences between primers 27 F-YM, 27 F-YM_MIX, and 341 F-NW.

Initial investigations found successful *C. trachomatis* amplification. However, sequencing failed to identify the microbe in oral microbiome samples. This may be attributed to the inability of Q-PCR analysis to produce qualitative data of sufficient sensitivity to detect *C*. *trachomatis* DNA [26–28]. Although it was anticipated that the 27 F-YM primer would fail to detect *C*. *trachomatis,* the additional failure of both the 341 F-NW and 27 F-YM (MIX) primers to identify this species was unexpected. The 27 F-YM (MIX) primer was expected to provide whole 16S rRNA sequencing. The use of this primer mix provided increased *C. trachomatis* identification due to the inclusion of 27 F-YM_Chl, specific to *Chlamydiales,* as well as the 341 F-NW primer previously demonstrated to increase accuracy in detection of *C*. *trachomatis* [16, 25]. The failure to detect *C. trachomatis* within patient samples highlights the need for a more stringent primer design for detection of this pathogenic microbe in laboratory settings. Additionally, utilising a different sequencing technology to assess the 16S rRNA gene—such as Pacific Biosciences technology (PacBio)—may provide extra insight and highlight potential pitfalls associated with ONT Nanopore sequencing and its primers.

In the bioinformatic pipeline comparisons performed in this study, the use of non-specific parameters using Porechop demonstrated significantly more bacterial species than the specific parameters across all participants. This was due to the significant decrease in accuracy in filtering, demultiplexing, and trimming of the sequencing data, resulting in a wide variety of data for subsequent analyses [29]. It is likely that the decreased specificity in adapter and barcode identification and filtering for the non-specific dataset resulted in incorrect barcode allocation [30]. As such, the use of sequence-specific adapters is vital for accurate DNA sequence identification, whilst also increasing the accuracy for downstream taxonomic classification [31, 32]. The specific Porechop parameters utilised publicly available raw data and demonstrated increased accuracy of sequence-specific adapter and barcode trimming, filtering, and demultiplexing on complex microbial samples. In addition, the use of specific parameter analysis in this study produced similar findings to Lüth et al., validating the bioinformatic approach as a reliable and effective tool for nanopore sequencing analysis. Use of the NanoCLUST pipeline using specific Porechop parameters matched Lüth et al. in species identification and the relative abundance of each microbe for a large proportion of participants (8/10 matched). Application of the EPI2ME pipeline failed to accurately identify the number of microbes and their relative abundance due to the large portion of reads assigned to unknown or other categories.

Analysis of vaginal microbiome samples by EPI2ME did not match the performance of NanoCLUST. This may be due to the EPI2ME pipeline classifying each read individually, while NanoCLUST clusters reads together to create a robust polished consensus sequence, then classified via BLAST [33]. The grouping of reads through NanoCLUST is thought to decrease the impact of sequencing errors and the creation of polished consensus sequences for taxonomic classification, further reducing errors and improving accuracy [33, 34]. For nanopore sequencing, this may be of more importance due to the limitations of the sequencing technology in reduced read accuracy when compared to other platforms [15]. With NanoCLUST demonstrating superior accuracy when paired with specific Porechop parameters, the pipeline was then applied to characterise the core vaginal microbiota composition across samples, examining both primer and bioinformatic performance of Nanopore sequencing data.

To investigate any specific considerations in vaginal microbiome swabs related to the sample site, 16S rRNA amplification and expected bacterial abundance, a pilot investigation of 12 healthy participants was completed. The data identified 9/12 samples to have concordant results across primer sets related to the dominant microbe, with 3/12 samples exhibiting discordance among the same sample using different primers. In samples 68 and 79, the 27 F-YM (MIX) primer identified *L. crispatus*; however, the 341 F-NW primer demonstrated *L. iners* as the dominant species in these samples.

Analysis of sample 36 showed *L. jensenii* dominance for primer 341 F-NW, with the 27 F-YM_MIX primer highlighting *L. mulieris* as the dominant microbe. This discordance may be attributed to the inability of 341 F-NW to amplify regions V1–V2, demonstrated to have the highest resolution for species and genus identification [35]. The difference in dominant species may also be the result of increased sequencing depth for the 341 F-NW primers, impacting the number of reads utilised for microbial profiling (Supplementary Table S1) [36]. The discordance between *L. iners* and *L. crispatus* may have resulted from an overestimation of *L. iners*, common when utilising 16S rRNA primers that align to different variable regions within the gene [37]. Interestingly, investigations by O’Callaghan et al. have shown that different *Lactobacillus spp*. are particularly prone to amplification bias based on the location of the forward and reverse primers within the 16S rRNA gene in short-read sequencing, affecting long-read sequencing of vaginal microbiome composition [37].

Additionally, distinguishing between *L. mulieris* and *L. jensenii* is exceptionally challenging due to their high sequence similarity, with only a two-nucleotide difference. An additional challenge resulted due to both the 27 F-YM (MIX) and 341 F-NW primers generating less than the average reads (~ 1,500 for 27 F-YM (MIX) and 2472 for 341 F-NW (Supplementary Table 1)), further contributing to the difficulty in distinguishing between these species. Ene et al. also highlight that distinguishing between the two species is not possible through short-read 16S rRNA sequencing [38]. Due to this, a metagenomics or species-specific sequencing approach is required to accurately discern which species is truly dominant in sample 36 [38]. Whilst our analysis identified different dominant microbes across the samples examined, all were a part of the *Lactobacillus* species, with most samples representing CST I.

This study aimed to optimise and refine 16S rRNA sequencing and bioinformatic approaches to accurately characterise the core vaginal microbiota in samples of healthy Australian reproductive-aged females. While quantification of *C*. *trachomatis* showed promise in the detection for varied *C*. *trachomatis* concentrations, particularly for the 27 F-YM (MIX) primer, the use of nanopore sequencing failed to detect *C*. *trachomatis* across all three primer pairs within oral microbiome samples. Further studies are required to optimise primer design and PCR protocols for *C*. *trachomatis* detection in full-length 16S rRNA sequencing.

The NanoCLUST analysis pipeline, coupled with the specific Porechop parameters, demonstrated superior accuracy in species detection and relative abundance than use of non-specific settings, closely aligning with the findings of Lüth et al. However, discrepancies between the Lüth et al. approach and the findings of this study require further benchmarking using known mock microbial community data to further critique and analyse the accuracy of the NanoCLUST pipeline when paired with the specific Porechop parameters. The absence of a mock community introduces challenges in precision assessments of each bioinformatic tool, as a known starting composition is lacking.

Finally, while the comparison of primers 341 F-NW and 27 F-YM_MIX provided relatively similar results among multiple vaginal microbiome samples, some discrepancies were noted in the dominant Lactobacillus species within the same samples. To improve the accuracy of microbial detection using Nanopore sequencing, a whole genome metagenomic sequencing approach is recommended, utilising adaptive sampling to reduce contamination from human host DNA in the samples. Additional known mock communities need to be used to assess overall precision and accuracy of each methodological approach. Additionally, further investigations comparing long-read sequencing technologies—such as PacBio and Nanopore—should be performed to identify the most accurate platform and provide insights into the most reliable technology for microbiome analysis.

## Supplementary Information

Below is the link to the electronic supplementary material.

Supplementary file1 (DOCX 783 KB)

## Data Availability

The datasets analysed during the current study are available from the corresponding author on reasonable request. The underlying code for this study is available in the Bioinformatic-Pipeline GIT repository and can be accessed via this link: https://github.com/izzydavidson/Bioinformatic-Pipeline.git.

## Materials availability

The datasets analysed during the current study are available from the corresponding author on reasonable request. The underlying code for this study is available in the Bioinformatic-Pipeline GIT repository and can be accessed via this link: https://github.com/izzydavidson/Bioinformatic-Pipeline.git.
