# Supplementary material for: Toward accurate vaginal microbiome profiling: protocol, bioinformatics, and core microbiota characterisation
Source: J Assist Reprod Genet. 2025 May 29;42(7):2421–36. doi: 10.1007/s10815-025-03509-2 (PMC12356782; doi:10.1007/s10815-025-03509-2)
Supplement: Supplementary file 1 — Supplementary file1 (DOCX 783 KB) [file 10815_2025_3509_MOESM1_ESM.docx]

**Toward Accurate Vaginal Microbiome Profiling: Protocol, Bioinformatics, and Core Microbiota Characterization**

**Supplementary Table S1.** Sequencing depth and quality for each sample ID and primer.

Table 1: Coverage of Reads Binned Per Sample Compared Across Primer 27F-YM_MIX and 341F-NW and Quality Scores after NanoFilt Filtering and After Porechop Demultiplexing and Adapter Trimming.

|  | 27F-YM_MIX Primer |  | 341F-NW Primer |  |
| --- | --- | --- | --- | --- |
| Patient ID | **Reads Binned** | **Quality** | **Reads Binned** | **Quality** |
| 01 | 2,763 | 18 | 3,872 | 15 |
| 13 | 1,625 | 18 | 3,575 | 15 |
| 18 | 2,519 | 18 | 3,322 | 15 |
| 23 | 1,729 | 18 | 2,685 | 15 |
| 36 | 1, 086 | 18 | 1,798 | 15 |
| 40 | 1,299 | 18 | 1,490 | 15 |
| 57 | 1,098 | 18 | 2,059 | 15 |
| 61 | 1,526 | 18 | 2,152 | 15 |
| 68 | 1,804 | 18 | 2,631 | 15 |
| 79 | 588 | 18 | 1,040 | 15 |
| 90 | 812 | 18 | 3,188 | 15 |
| 99 | 1,963 | 18 | 1,852 | 15 |
| Average | 1,567 (MIN = 578, MAX = 2, 763) | 18 | 2472 (MIN = 1,040, MAX = 3,872) | 15 |


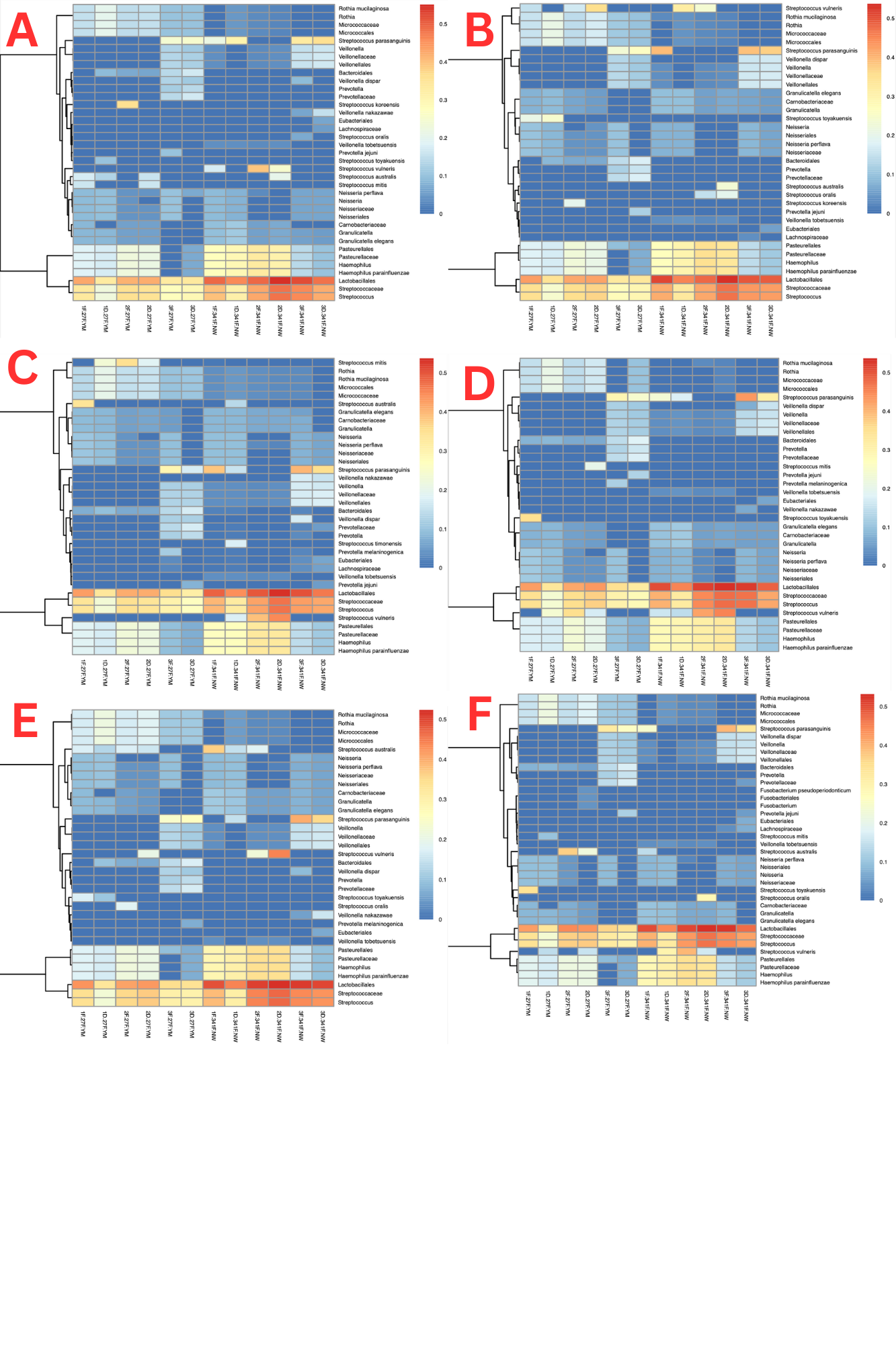
**Supplementary Figure S2.** Comparison of bacterial abundance for different Base Calling and Porechop Methodologies.

Figure 5.2: Heatmap of Bacterial Abundance and Detection for each DNA Sample (1F, 1D, 2F, 2D, 3F and 3D) for Dorado Simplex Base Calling with Either (A) Porechop Non-specific or (B) Specific Parameters, Dorado Duplex Base Calling with Either (C) Non-specific or (D) Specific Porechop Parameters and GUPPY Base Calling for Either (E)Specific or (F) Non-specific Porechop Adapter and Barcode Trimming and Demultiplexing Parameters. Created in R Studio (rstudio-education.github.io)
